# Transfer of Soleus Muscular Branch of Tibial Nerve to Deep Fibular Nerve to Repair Foot Drop After Common Peroneal Nerve Injury: A Retrospective Study

**DOI:** 10.3389/fneur.2022.745746

**Published:** 2022-02-11

**Authors:** Bingbo Bao, Haifeng Wei, Hongyi Zhu, Xianyou Zheng

**Affiliations:** Department of Orthopedic Surgery, Shanghai Jiao Tong University Affiliated Sixth People's Hospital, Shanghai, China

**Keywords:** common peroneal nerve injury, tibial nerve, nerve transfer, foot drop, reconstruction

## Abstract

**Objective:**

Common peroneal nerve (CPN) injury that leads to foot drop is difficult to manage and treat. We present a new strategy for management of foot drop after CPN injury. The soleus muscular branch of the tibial nerve is directly transferred to the deep fibular nerve, providing partial restoration of motor function.

**Methods:**

We retrospectively reviewed eight patients treated for CPN injury between 2017 and 2019. The soleus muscular branch of the tibial nerve was transferred to the deep fibular nerve to repair foot drop. Electrophysiology was conducted, and motor function was assessed. Motor function was evaluated by measuring leg muscle strength during ankle dorsiflexion using the British Medical Research Council (BMRC) grading system and electromyography (EMG).

**Results:**

In 10–15 months postoperatively, EMG revealed newly appearing electrical potentials in the tibialis anterior, extensor hallucis longus, and extensor toe longus muscle (*N* = 7). Two patients achieved BMRC grade of M4 for ankle dorsiflexion, 2 patients achieved M3, 1 patient achieved M2, and 2 patients achieved M1. Four patients showed good functional recovery after surgery and could walk and participate in activities without ankle-foot orthotics.

**Conclusion:**

Surgical transfer of the soleus muscular branch of the tibial nerve to the deep fibular nerve after CPN injury provides variable improvements in ankle dorsiflexion strength. Despite variable strength gains, 50% of patients achieved BMRC M3 or greater motor recovery, which enabled them to walk without assistive devices.

## Introduction

Common peroneal nerve (CPN) injury is the most frequently observed lower limb nerve injury (1–3). CPN injury often led to motor and sensory dysfunction in the areas it innervates; for example, loss of ankle dorsiflexion due to the affected tibialis anterior, foot eversion due to the affected peroneus longus and brevis, and dorsal foot sensory loss (4). For patients with CPN injury, loss of motor function results in foot drop, which is characterized by a foot slap as the heel strikes the pavement and a steppage gait (5). In CPN injuries, foot drop is the main symptom that affects quality of life.

At present, many methods are available for treating foot drop caused by CPN injury. Traditional treatments include ankle-foot orthoses (AFO) (3, 6), nerve exploration and neurolysis (4), autologous nerve grafting (7), and functional orthopedic surgery (8). Although these methods have helped to restore function to a certain extent in these patients, each has limitations. AFOs, for example, can aid patients' walking, but patients are usually unsatisfied because the device is uncomfortable, difficult to keep clean, and mobility is still limited (9). Nerve exploration and neurolysis usually has no clear therapeutic purpose and no long-lasting repair (10). Allographic nerve grafting is rarely successful when performed more than 6 months after injury, or if the grafts are longer than 6 cm (3, 11, 12). Therefore, for CPN defects longer than 6 cm or for treatment delays >6 months, nerve grafting may not be the optimal treatment. Another commonly used treatment for foot drop is tendon transfer (13–15). Although tendon transfer successfully restores ambulatory function without assistive devices in some patients, this procedure cannot completely restore normal gait, and dorsiflexion remains weak (16). Tendon transfer is also fraught with long-term complications, including flat foot deformity, arthritis, and hindfoot valgus deformity (17). In general, therefore, the current regimens for treating foot drop caused by CPN injury are not ideal and pose huge challenges for surgeons.

Nerve transfers are widely used to restore upper-limb function and have achieved good therapeutic effects (18). However, nerve transfers are used less often to repair lower-limb nerve injuries. Previous anatomic studies have demonstrated that use of proximal tibial nerve branches as grafts for the peroneal nerve are feasible alternatives for restoring ankle dorsiflexion (19, 20). Whether this kind of nerve transfer would improve strength and/or function in patients is unknown. However, one clinical study obtained good results with a partial tibial nerve transfer to the tibialis anterior motor branch for treatment of a peroneal nerve injury (21). Taken together, these findings prompted us to develop a new strategy for the management of CPN-related foot drop through nerve transfer. Specifically, we used the soleus muscular branch of the tibial nerve as the donor for the deep fibular nerve after CPN injury, and then evaluated functional outcomes using the BMRC muscle strength grading system (22) and EMG.

## Methods

### Patient Information

We retrospectively reviewed the medical charts and EMG records of 8 patients who underwent nerve transfers for the treatment of peroneal nerve injuries from January 2017 to December 2019. We received Hospital Ethics Committee approval to conduct this retrospective study (23). Informed consent was also obtained from each patient, and the study was carried out in accordance with Declaration of Helsinki (24).

All subjects had high-energy trauma to the knee joint without open wounds. After the trauma event, foot drop gradually appeared and noted by patients, and CPN injury was diagnosed after ultrasound and EMG examination. Two patients had undergone CPN exploration surgery at other hospitals, but their recovery was poor. All 8 subjects failed to seek prompt treatment for their CPN injury, instead waiting more than 6 months following injury before seeking further medical treatment.

Preoperative evaluation for nerve transfer included clinical examination of foot strength and standard EMG recordings, which invariably revealed complete paralysis and denervation of the muscles innervated by the CPN. Normal function of the tibial nerve was observed in all subjects. BMRC grading was used to evaluate ankle dorsiflexion strength preoperatively. The BMRC grading system rates muscle strength on a scale of M0 (no muscle contraction) to M5 (normal muscle contraction against full resistance) (22). At the time of preoperative evaluation, all patients were graded M0. Indications for nerve transfer surgery included any closed, high-energy trauma that failed to show clinical or EMG evidence of CPN recovery 6 months after injury. Contraindications for nerve transfer surgery included dysfunction or paralysis of the tibial nerve, pre-existing peripheral neuropathy, major posterior compartmental injury, or any other lower extremity nerve injuries. All patients underwent nerve grafting in which the soleus muscular branch of the tibial nerve served as a donor nerve that was transferred and anastomosed to the deep fibular nerve.

We collected serial postoperative information, such as results of clinical examination of the patient's foot strength and EMG results. Clinical outcomes we assessed were strength of muscles involved in ankle dorsiflexion and toe extension. Muscle strength was evaluated again with the BMRC grading system. The results of the nerve transfer were categorized as poor when muscle strength was judged to be grade M2 or less and as good when the muscle strength was graded M3 or M4 (22). EMG recordings were used to assess CPN and tibial nerve function and the muscles they innervate 1 year after nerve-transfer surgery.

### Surgical Nerve Transfer Procedure

**Figure 1:**
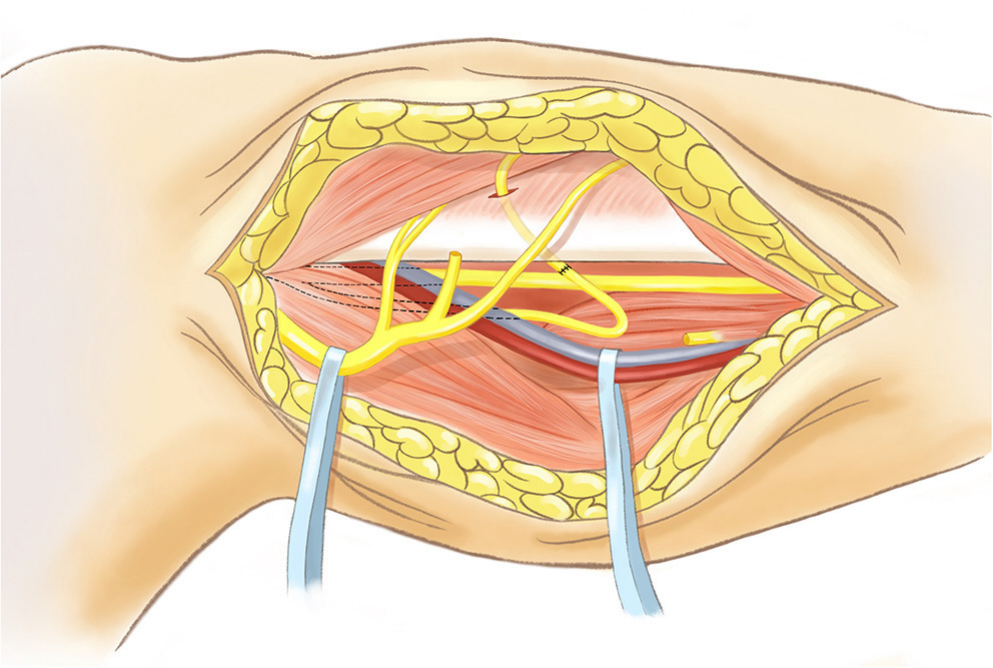
Schematic diagram of right leg (medial view) showing surgical isolation of the soleus muscular branch of the tibial nerve and its subsequent transfer to the deep fibular nerve. Retractors are indicated in blue, one on CPN, and one on deep vessels. Axotomy of the soleus muscular branch of the tibial nerve is indicated, and its anastomoses to deep fibular nerve is indicated by black stich pattern. Diagram is based on an illustration created by the Mayo Foundation for Medication Education and Research reproduced and modified. All rights reserved by the original copyright holder.

An overview of the surgical procedure is schematically illustrated in Figure 1. Briefly, the patient was placed in supine position, and the procedure was performed under the guidance of an operating microscope. A curvilinear incision was made laterally on the injured leg, starting at the level of the popliteal fossa and ending 5 cm below the fibular head. Initially, layered dissection was performed to identify the main trunk of the CPN at the top of the popliteal fossa and to identify distal divisions of the CPN, including deep and superficial peroneal nerves. After isolating the deep peroneal nerve, we cut the deep peroneal nerve tissue. We then released the peroneal tunnel created by the upper edge of the fibula in order to provide space for the distal end of the deep peroneal nerve to pass through and to encourage correct axonal regrowth.

Next, dissection was performed to identify the main trunk of the tibial nerve located under the fibula and the soleus muscular branch of the tibial nerve located above the soleus muscle. After isolating the soleus muscular branch of the tibial nerve, we cut the soleus muscular branch of the tibial nerve. Finally, we performed tension-free coaptation between the proximal end of the soleus muscular branch of the tibial nerve and the distal end of the deep peroneal nerve.

During the procedure, we used electrical stimulation to confirm normal nerve function. Postoperatively, the limb was kept immobilized for 3 weeks. This was followed by an intensive programme of physiotherapy, which included motor re-education and strengthening exercises, such as use of orthotic devices in daily life, adjuvant drug therapy, electrical stimulation, and so on.

## Results

### Group Results

Detailed patient information is shown in Table 1. Of the eight patients, two were females and six were males, with an average age of 40 years (range, 24–58 years). All patients underwent the entire follow-up regimen; the average follow-up duration was 24 months (range, 13–38 months). We lost no subjects to follow-up. No intraoperative or postoperative complications occurred.

**Table 1:** Demographics of patients with CPN injury who received nerve transfer, and selected results of clinical assessment.

**Patient number**	**Age yr/gender**	**Injury type**	**Time to surgery (mo)**	Preoperative BMRC^*^dorsiflexion/5	**Preoperative EDX (±)**	**Time after surgery (mo)**	Postoperative BMRC^*^dorsiflexion	**Preoperative EDX (±)**
1	44/M	Crush	8	0	TA(–); EHL(–); ETL(–); So(+)	18	M4	TA(+); EHL(+); ETL(+); So(–)
2	30/M	Fall	6	0	TA(–); EHL(–); ETL(–); So(+)	38	M1	TA(+); EHL(–); ETL(–); So(+)
3	24/F	Fall	7	0	TA(–); EHL(–); ETL(–); So(+)	30	M1	TA(+); EHL(+); ETL(–); So(–)
4	45/M	Crush	6	0	TA(–); EHL(–); ETL(–); So(+)	25	M4	TA(+); EHL(+); ETL(+); So(+)
5	39/F	Crush	58	0	TA(–); EHL(–); ETL(–); So(+)	28	M0	TA(–); EHL(–); ETL(–); So(–)
6	47/M	Crush	6	0	TA(–); EHL(–); ETL(–); So(+)	22	M3	TA(+); EHL(+); ETL(–); So(–)
7	33/M	Fall	11	0	TA(–); EHL(–); ETL(–); So(+)	18	M2	TA(+); EHL(+); ETL(–); So(+)
8	58/M	Crush	8	0	TA(–); EHL(–); ETL(–); So(+)	13	M3	TA(+); EHL(+); ETL(+); So(–)

*M, male; F, female; BMRC, british medical research council; EDX, electrodiagnostic examination; TA, tibialis anterior; EHL, extensor hallucis longus; ETL, extensor toe longus; So, soleus; EMG results: ±, no electrical activity/newly acquired action potentials in tibialis anterior, extensor hallucis longus, or extensor toe longus*.

*The sample size was too small to conduct meaningful statistical analysis*.

* *BMRC muscle strength grading scale: M0, no muscle contraction; M1, trace contraction; M2, active movement with gravity eliminated; M3, active movement against gravity; M4, active movement against gravity and resistance; M5, normal muscle strength (22)*.

In all patients, tibial nerve soleus muscle branches and deep peroneal nerves were successfully joined directly *via* tension-free coaptation. Postoperative EMG examination showed previously undetectable compound action potentials in the tibialis anterior, extensor hallucis longus, and extensor toe longus of all but one patient, indicating that the tibial nerve soleus muscle branch had regenerated and connected to the deep peroneal nerve. In three patients, EMG recordings also demonstrated innervation of the soleus muscle, illustrating that the soleus muscle was innervated by multiple nerves. To sum up, after nerve transfer surgery, four of the eight patients (50%) achieved an ankle dorsiflexion BMRC grade of M3 or greater, indicating motor recovery of ankle dorsiflexion, enabling them to ambulate without assistive devices.

Of the eight patients, two achieved an ankle dorsiflexion BMRC grade of M4, two had a grade of M3, one had a grade of M2, and two had a grade of M1. Only one patient remained at grade M0 and failed to regain any observable muscle activity. Four patients did not wear an AFO postoperatively, but the other four patients still required external orthoses after rehabilitation.

### Typical Case

A male patient was diagnosed with CPN injury following a crush injury 8 months prior to examination and treatment at our hospital. Preoperatively, ultrasound confirmed the continuity of the CPN and severe compression of the CPN scar. EMG recordings confirmed that the CPN was completely injured and that the tibial nerve was intact. Physical examination revealed foot drop and completely diminished ankle dorsiflexion function.

**Figure 2:**
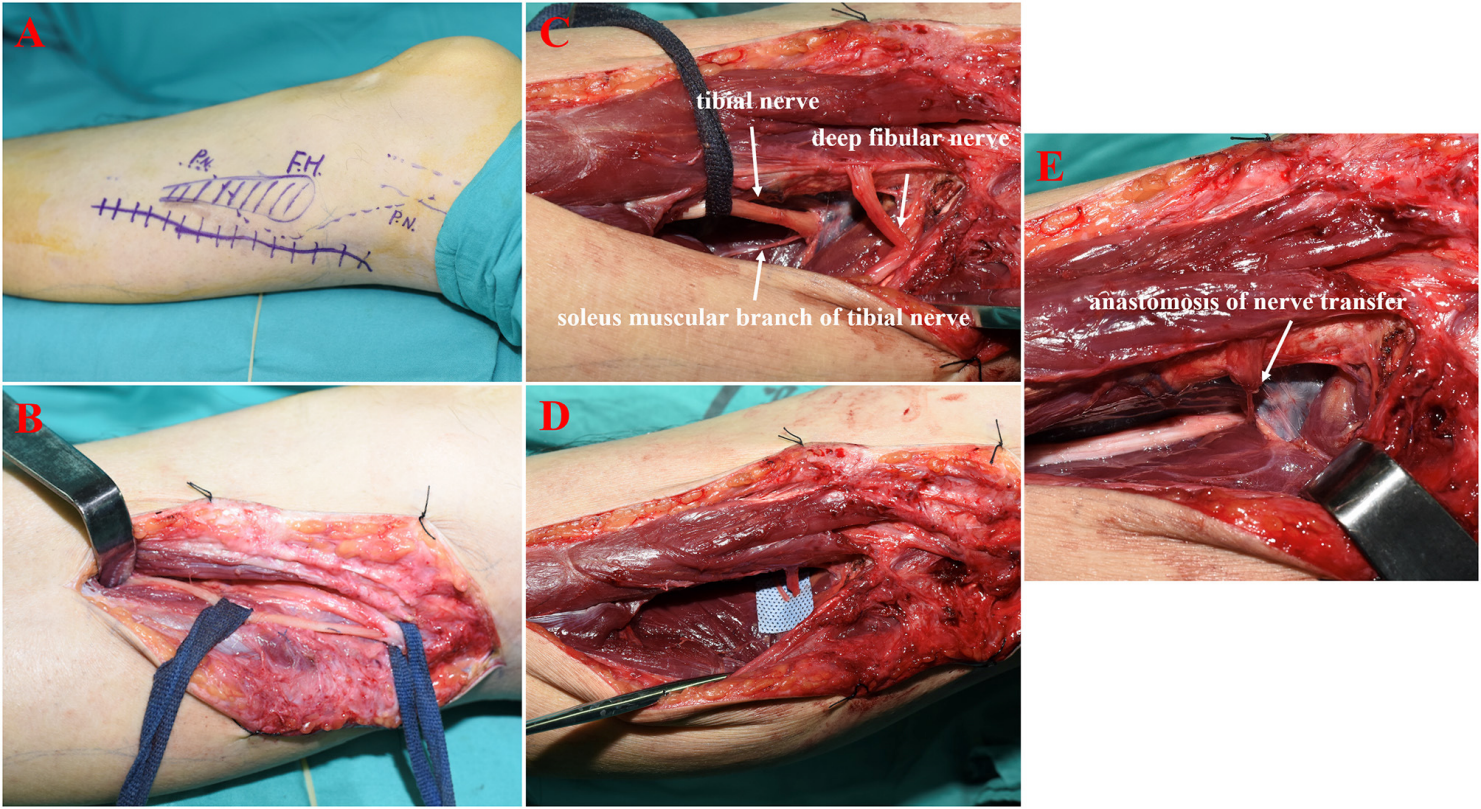
Pre- and intraoperative photographs of a patient who had undergone surgical transfer of the soleus muscular branch of the tibial nerve to the deep peroneal nerve. **(A)** Preoperative planning showing the schematic diagram of the incision site. **(B)** Isolation and exposure of the deep peroneal nerve. **(C)** Isolation and exposure of the soleus muscular branch of the tibial nerve. **(D)** Exposure of the distal end of the severed deep peroneal nerve and the proximal end of the soleus muscular branch of the tibial nerve. **(E)** Tension-free coaptation of nerve stump.

**Figure 3:**
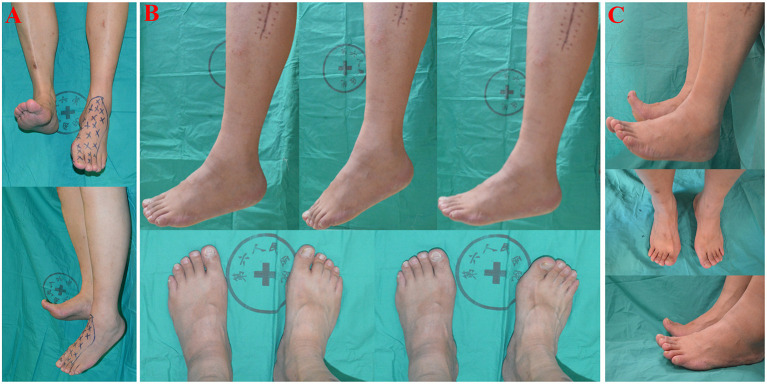
**(A)** Patient had significant foot drop of the left foot after common peroneal nerve injury (Frontal view and Lateral view). Blue-colored indicators drawn on the patient's skin are a reference anesthetic area. **(B)** Postoperative results of 6 months after nerve-transfer surgery. Left foot is in neutral position, plantarflexion, and dorsiflexion position without weights. Both feet are in plantarflexion and dorsiflexion position with weights. **(C)** Postoperative results of 12 months after nerve-transfer surgery. Both feet are in dorsiflexion with or without weights.

To address the patient's difficulty in walking caused by foot drop, we decided to repair the CPN by performing nerve transfer surgery, with the soleus muscular branch of the tibial nerve serving as the donor and the deep fibular nerve as the recipient, as described above and shown in Figure 2. Following surgery, the patient's limb remained immobilized for 3 weeks. This initial period was followed by an intensive programme of physiotherapy, which included motor re-education and strengthening exercises. The injured limb was graded as BMRC M0 before treatment (Figure 3A). At the 6-month postoperative follow-up, the patient's ankle dorsiflexion BMRC grade was M3 (Figure 3B), and at the 12-month postoperative follow-up, his BMRC grade was M4 (Figure 3C). The patient was satisfied with the treatment and now can walk normally without using an AFO (Supplementary Video 1).

## Discussion

CPN injury remains very common in clinical practice, but in terms of injury mechanism, closed CPN injuries occupy a major proportion. These injuries are usually due to high-energy trauma to the knee joint and include crush, strain, and violent rotation injuries (25). As surgical technology and treatment capabilities have continued to improve, the cure rate for CPN injuries has also improved (3, 26). However, lower-limb nerve injuries often fail to attract enough attention from patients and doctors, which often leads to delayed or inadequate treatment.

As patients with CPN injuries do not present with obvious visual manifestations, closed CPN injuries, in particular, are often treated conservatively, thus delaying attempts at surgical repair. If a damaged CPN is not treated in a timely manner, often the outcome is poor. For many patients with closed CPN injury, by the time they visit a specialist clinic for treatment of their foot drop, unfortunately, they have already missed the optimal timing for treatment through nerve repair. Equally important, there is no unified with good functional recovery treatment for patients with delay CPN injury (27).

Over the last decade, many studies have shown that nerve transfer is a feasible way to restore limb function after nerve injury (28). Nerve transfer was also performed for repair of CPN injury (29–31). However, previous studies have shown highly contradictory results. The contradictory results may be related to the following reasons. Firstly, nerve transfer still requires nerve anastomosis, but at present, a considerable number of patients cannot achieve good recovery results after nerve anastomosis (32). Furthermore, central plasticity after CPN injury with gait change also hinders the process of nerve rehabilitation to a certain extent (33). Of course, the surgical method itself also affects the results to some extent, such as different donor nerves and the number of donor nerves (34). In the present study, although not all patients achieved good functional recovery, we still believe that this surgical method has solved, to a certain extent, the problem of reasonably treating CPN injury.

We believe that the surgical method we describe here has several advantages. Firstly, this new surgical strategy offers patients with certain kinds of CPN injuries (CPN damage longer that 6 cm) and who have delayed treatment (>6 months after injury) a new option for treatment (35). Although for some patients the recovery is not completely satisfactory, tendon transfer can still be used to repair foot drop. Secondly, our surgical strategy is a typical neuromotor branch coaptation with a motor branch, which provides the patient with a new source of motor nerve to regain muscle power. Compared with partial tibial nerve transfer, the soleus muscle branch transfer provides more reliable nerve recovery opportunities. At the same time, compared with autologous nerve transplantation approaches, this new surgical strategy only has one nerve coaptation, which can provide a more reliable opportunity for nerve recovery. Thirdly, the use of the soleus muscular branch of the tibial nerve transfer can partially weaken the ankle plantarflexion strength and create favorable conditions for the recovery of the ankle dorsiflexion function. However, our postoperative follow-up EMG results showed that three patients also had innervation of the soleus muscle, which may be related to the multi-branch innervation of the soleus muscle. This outcome also likely means that this procedure is less damaging to the donor site. Finally, this new surgical method is simple to learn and carry out, and the total operation time is short. The most important step is to find and isolate the donor nerve branch that needs to be transferred. Therefore, the technique should be relatively accessible to many surgeons.

This study also has some limitations. Firstly, the sample size of the study was too small, and more patients are needed to further verify the effectiveness of this procedure. Secondly, there may be large bias in treatment choices. Therefore, further clinical randomized control trial research may improve the evidence for better clinical treatment. Thirdly, the soleus muscular branch of the tibial nerve does not match the deep peroneal nerve particularly well in some patients, which may also be the reason for the less effective repair in some patients. Thus, we believe that this multi-branch nerve transfer repair approach to manage CPN injury and foot drop warrants further study. Finally, the criteria for case enrolment still needs to be further improved. Only with more precise surgical indications, patients may be able to obtain better treatment results.

## Conclusion

For optimal treatment of CPN injury, early detection and early treatment is clearly the best choice. However, for CPN injury that have gone untreated for >6 months, transfer of the soleus muscular branch of the tibial nerve to the deep fibular nerve may be a good treatment option. With further study, we may gain more evidence that this surgical repair technique appreciably improves foot drop of patients combined with good functional recovery.

## Data Availability Statement

The raw data supporting the conclusions of this article will be made available by the authors, without undue reservation.

## Ethics Statement

The studies involving human participants were reviewed and approved by Ethics Committee of Affiliated Sixth People's Hospital of Shanghai Jiao Tong University of China. The patients/participants provided their written informed consent to participate in this study.

## Author Contributions

XZ was responsible for study design and manuscript revision. BB and HW were responsible for data collection and analysis. BB was responsible for manuscript writing. HZ revised the manuscript. All authors critically reviewed the content of the manuscript, read, and approved the final manuscript.

## Funding

This study was supported by National Natural Science Foundation of China (Grant number: 81974331).

## Conflict of Interest

The authors declare that the research was conducted in the absence of any commercial or financial relationships that could be construed as a potential conflict of interest.

## Publisher's Note

All claims expressed in this article are solely those of the authors and do not necessarily represent those of their affiliated organizations, or those of the publisher, the editors and the reviewers. Any product that may be evaluated in this article, or claim that may be made by its manufacturer, is not guaranteed or endorsed by the publisher.

## Supplementary Material

Supplementary Video 1 The video shows the patient's recovery of ankle function and walking function 1 year after surgery.
